# Antibiotic use and quality indicators of antibiotic prescription in Bhutan: a point prevalence survey using the Australian National Antimicrobial Prescribing Survey tool

**DOI:** 10.1093/jacamr/dlad100

**Published:** 2023-08-22

**Authors:** Pem Chuki, Thinley Dorji, Rodney James, Khando Wangchuk, Sonam Yangzom, Yangchen Dema, Sangay Wangchuk, Dorji Wangdi, Tshering Deki, Chandra Limbu, Kuenzang Rangdel Dorji, Sonam Wangda, Kirsty Buising, Karin Thursky

**Affiliations:** Antimicrobial Stewardship Unit, Jigme Dorji Wangchuck National Referral Hospital, Thimphu, Bhutan; Department of Internal Medicine, Central Regional Referral Hospital, Gelephu, Bhutan; Department of Infectious Diseases, National Centre for Antimicrobial Stewardship, University of Melbourne, Melbourne, Victoria, Australia; Guidance Group, The Royal Melbourne Hospital, Melbourne, Victoria, Australia; Antimicrobial Stewardship Unit, Jigme Dorji Wangchuck National Referral Hospital, Thimphu, Bhutan; Department of Internal Medicine, Eastern Regional Referral Hospital, Monggar, Bhutan; Department of Internal Medicine, Eastern Regional Referral Hospital, Monggar, Bhutan; Department of Internal Medicine, Central Regional Referral Hospital, Gelephu, Bhutan; Department of Pharmacy, Central Regional Referral Hospital, Gelephu, Bhutan; Antimicrobial Stewardship Unit, Jigme Dorji Wangchuck National Referral Hospital, Thimphu, Bhutan; Antimicrobial Stewardship Unit, Jigme Dorji Wangchuck National Referral Hospital, Thimphu, Bhutan; Department of Pharmacy, Phuentsholing General Hospital, Phuentsholing, Bhutan; Antimicrobial Resistance Prevention Program, Ministry of Health, Thimphu, Bhutan; Victorian Infectious Diseases Service, The Royal Melbourne Hospital, Melbourne, Victoria, Australia; Department of Internal Medicine, Central Regional Referral Hospital, Gelephu, Bhutan; Department of Infectious Diseases, National Centre for Antimicrobial Stewardship, University of Melbourne, Melbourne, Victoria, Australia

## Abstract

**Background:**

The National Action Plan on Antimicrobial Resistance in Bhutan promotes the rational use of antibiotics. It is important to establish baseline data on the use of antibiotics and the quality indicators of antibiotic prescriptions to identify where improvement efforts may need to be focused.

**Objectives:**

To describe the prevalence and patterns of antibiotic prescription and establish baseline data regarding quality indicators of antibiotic prescriptions in four major hospitals in Bhutan.

**Methods:**

This was a point prevalence survey of antibiotic use among inpatients in June 2022 conducted using the Australian National Antibiotic Prescribing Survey (NAPS).

**Results:**

There were 314 patients (41.5%) receiving at least one antibiotic on the audit day. Among prescriptions reviewed, 278 (88.5%) had indications for use documented, 102 (32.5%) had a review or stop date documented and 120 (38.2%) had microbiology samples collected prior to antibiotics. Ceftriaxone (68; 21.7%), cefazolin (41; 13.1%) and metronidazole (32; 10.2%), were the common antibiotics prescribed. The most common indications for use were surgical prophylaxis (42; 13.4%), community-acquired pneumonia (39; 12.4%) and sepsis (26; 8.3%). There were 125 prescriptions (39.8%) that were compliant with national/therapeutic antibiotic guidelines and 169 (53.8%) where antibiotic prescriptions were appropriate.

**Conclusions:**

This study identified key areas for targeted interventions in antimicrobial stewardship programmes in Bhutan. The prevalence of antibiotic use, indications for use, and drug choices were similar to data from other countries. Documentation plans for durations of use, prolonged surgical prophylaxis and concordance of choices with guideline recommendations present opportunities for improvement.

## Introduction

Antimicrobial resistance (AMR) has become a major threat to global health and was associated with an estimated 4.95 million deaths in 2019.^1^ At the regional level, the highest all-age death rate attributable to AMR was in sub-Saharan Africa, followed by South Asia.^1^ Given the rapid global spread of multi- and pan-resistant bacteria that are not treatable with existing antimicrobials, the WHO has declared AMR as one of the top 10 global public health threats facing humanity.^2^ Efforts towards reducing the burden and costs associated with AMR requires a multisectoral approach. One common factor that is attributed to rising rates of AMR is the inappropriate and excessive use of antibiotics.^2,3^ Antibiotic use and antibiotic prescription are influenced by many factors that can collectively be addressed through antimicrobial stewardship initiatives.^4,5^

Measuring, monitoring and evaluating antibiotic prescribing practices in hospitals is important to ensure that context-specific stewardship initiatives are developed and implemented effectively. There are several available tools to do this, including point prevalence survey protocols from the WHO.^6,7^ The National Antimicrobial Prescribing Survey (NAPS) is a voluntary web-based qualitative auditing platform that has been adopted by over 75% of all public and private facilities in Australia since its release in 2013 and successfully used in Canada, New Zealand, the UK, Fiji, Malaysia, Papua New Guinea, Timor-Leste and Vietnam.^8^

Bhutan is a country with a population of 0.7 million situated in the eastern Himalayas. It has universal access to free public healthcare, and health policies are centrally regulated by the Ministry of Health. The country has demonstrated a strong commitment to AMR-related policies and activities. Bhutan endorsed the National Action Plan on Antimicrobial Resistance in 2017 with a One Health focus.^9^ National antimicrobial prescribing guidelines were developed and endorsed in 2018 and implemented across all hospitals.^10^

Antimicrobial stewardship programmes were established at the Jigme Dorji Wangchuck (JDW) National Referral Hospital, Thimphu in 2016 and at the Central Regional Referral Hospital in Gelephu, Eastern Regional Referral Hospital in Monggar and Phuentsholing General Hospital in 2019. These four hospitals were designated as sentinel sites for the surveillance of antimicrobial use under the Fleming Fund project. The Fleming Fund is a UK Aid programme that supports low- and middle-income countries to generate, share and use data to improve antimicrobial use and encourage investments in AMR. The antimicrobial stewardship programme at the JDW National Referral Hospital is delivered by a team that includes a clinical microbiologist, a pharmacologist, physicians and nurses. The antimicrobial stewardship programmes in the other hospitals are delivered by physicians, nurses and pharmacists. Some of the antimicrobial stewardship interventions in these hospitals include education and training of staff, an approval system to manage access to restricted broad-spectrum antibiotics, surveillance of antimicrobial consumption and dedicated audits of specific antibiotic use.

This study was conducted hospital-wide to obtain baseline information on antibiotic prevalence, the most common antibiotics prescribed, the appropriateness of use as per national guidelines and quality indicators of antibiotic prescriptions in the four major hospitals in Bhutan. The aim was to identify areas for focused interventions to optimize the use of antibiotics in hospitals and contribute to efforts towards preventing and reducing AMR in Bhutan.

## Methods

### Study design and setting

This was a point prevalence survey of antibiotic use in four major hospitals in Bhutan: JDW National Referral Hospital (380 beds) in Thimphu, Central Regional Referral Hospital (150 beds) in Gelephu, Eastern Regional Referral Hospital (150 beds) in Monggar and Phuentsholing General Hospital (60 beds).

Bhutan has a three-tiered healthcare system with 10-bed hospitals at the primary level, district and general hospitals at the secondary level and three referral hospitals at the tertiary level.^11^ The JDW National Referral Hospital, Central Regional Referral Hospital and Eastern Regional Referral Hospital provide tertiary-level care and serve as strategic referral points in their region. They are equipped with specialist services such as ICUs, haemodialysis units and surgical services. These three hospitals are designated as teaching hospitals with intern medical doctors and postgraduate trainees among the prescribers. Phuentsholing General Hospital is a secondary-level hospital but is equipped with blood culture, haemodialysis and surgical services as it caters to a relatively larger population. The JDW National Referral Hospital caters to the largest patient load, followed by the Central Regional Referral Hospital. The number of doctors and specialists posted is highest at the JDW National Referral Hospital. While patients are expected to follow a referral pathway of accessing care at the primary-level hospital followed by the designated district/general hospital, it is often a common finding that patients jump the referral pathway and present directly at the tertiary-level hospitals.

### Study population

The data were collected between 2 and 18 June 2022. All inpatients admitted at 8 am on the day of the audit were eligible. The patients admitted for day-care procedures were excluded. The patients’ medical records were reviewed to capture patients on current antibiotics and single-dose antibiotics administered within the previous 24 h.

### Study tool

This study adopted the NAPS tool that was developed by the National Centre for Antimicrobial Stewardship, Australia, in 2013.^8^ It is an online auditing platform with a standardized point prevalence survey suitable for use in all hospitals to support real-time data collection and reporting with dashboards and aggregation of data for benchmarking. Prescriptions of topical antimicrobials, systemic antifungals, systemic antivirals and antimycobacterial agents were not included in auditing for this survey.

### Data collection

The survey was conducted by 12 local auditors in Bhutan (3 physicians, 4 pharmacists and 5 Infection Prevention and Control nurses). The auditors were trained on the NAPS data collection protocol and on the assessment of ‘compliance with guidelines’ and ‘appropriateness’ by Australia-based staff. According to the NAPS protocol,^6^ antimicrobial prescription is initially recorded as follows: ‘optimal’ if prescription follows therapeutic guidelines or has been reviewed by an infectious diseases clinician or clinical microbiologist; ‘adequate’ if the prescription does not optimally follow therapeutic guidelines but a reasonable alternative choice of antimicrobial is selected; ‘suboptimal’ if the choice of antibiotic is unreasonable or there is an allergy mismatch; ‘inadequate’ if the choice is unlikely to treat the likely causative agent; or it may be ‘not assessable’ if information such as indication is not documented. Those prescriptions recorded as ‘optimal’ or ‘adequate’ are categorized as ‘appropriate’ and those that are recorded as ‘suboptimal’ or ‘inadequate’ are categorized as ‘inappropriate’. In our study, the National Antibiotic Guideline^10^ was taken as the standard therapeutic guideline for the country.

When there were any difficulties in performing assessments during data collection, clarifications and support were provided by the focal person for the antimicrobial stewardship programme at the JDW National Referral Hospital and difficulties were further discussed with the NAPS staff in Australia.

### Data analysis

The data collected were de-identified and entered in the NAPS online database by the auditors.^6^ The data were extracted into Microsoft Excel and the variables of interest were analysed. Categorical data are presented as frequencies and percentages and continuous variables are summarized as mean and standard deviation.

### Ethics

This study was conducted according to the principles of the Declaration of Helsinki. Administrative approval was obtained from the Policy and Planning Division, Ministry of Health Bhutan, and the hospital administrators of the participating sites.

## Results

There were 756 patients captured during the study period: 487 at the JDW National Referral Hospital, 78 at the Central Regional Referral Hospital, 112 at the Eastern Regional Referral Hospital and 79 at Phuentsholing Hospital. There were 314 patients (41.5%) patients receiving at least one antibiotic on the audit day: 206 (42.3%) at JDW National Referral Hospital, 42 (53.8%) at Central Regional Referral Hospital, 38 (33.9%) at Eastern Regional Referral Hospital and 28 (35.4%) at Phuentsholing General Hospital. The basic description of the patients and antibiotic use in the four hospitals is shown in Table 1.

**Table 1.** Description of the prescriptions and their quality indicators audited using the NAPS in four major hospitals in Bhutan, June 2022

Variables	Overall (314)		JDWNRH (206)		CRRH (42)		ERRH (38)		PGH (28)	
	*n*	%	*n*	%	*n*	%	*n*	%	*n*	%
Sex										
Female	148	47.1	95	46.1	21	50.0	20	52.6	12	42.9
Male	166	52.9	111	53.9	21	50.0	18	47.4	16	57.1
Speciality departments										
General medicine	108	34.4	67	32.5	10	23.8	7	18.4	24	85.7
General surgery	67	21.3	41	19.9	17	40.5	9	23.7	—	—
Paediatrics	38	12.1	28	13.6	5	11.9	5	13.2	—	—
Orthopaedic surgery	35	11.1	23	11.2	2	4.8	10	26.3	—	—
Intensive/critical care	22	7.0	13	6.3	3	7.1	6	15.8	—	—
Gynaecology	17	5.4	9	4.4	4	9.5	1	2.6	3	10.7
Ear/nose/throat surgery	11	3.5	11	5.3	—	—	—	—	—	—
Obstetrics	9	2.9	8	3.9	—	—	—	—	1	3.6
Dermatology	3	1.0	3	1.5	—	—	—	—	—	—
Neonatology	2	0.6	2	1.0	—	—	—	—	—	—
Oncology	1	0.3	1	0.5	—	—	—	—	—	—
Ophthalmology										
History of drug allergy										
Present	4	1.3	1	0.5	1	2.4	2	5.3	—	—
Absent	218	69.4	151	73.3	39	92.9	25	65.8	3	10.7
Not documented	92	29.3	54	26.2	2	4.8	11	28.9	25	89.3
Microbiological sample collected prior to antibiotics	120	38.2	78	37.9	20	47.6	20	52.6	2	7.1
Route										
IV	248	79.0	158	76.7	32	76.2	36	94.7	22	78.6
Oral	66	21.0	48	23.3	10	23.8	2	5.3	6	21.4
Indication documented	278	88.5	181	87.9	38	90.5	36	94.7	23	82.1
Review or stop date	102	32.5	61	29.6	24	57.1	15	39.5	2	7.1
Number of antibiotics										
One	190	60.5	126	61.2	28	66.7	25	65.8	11	39.3
Two	112	35.7	73	35.4	10	23.8	13	34.2	16	57.1
Three	12	3.8	7	3.4	4	9.5	—	—	1	3.6
Compliance with guideline										
Compliant with guideline	125	39.8	63	30.6	29	69.0	26	68.4	7	25.0
Directed therapy^a^	26	8.3	21	10.2	1	2.4	3	7.9	1	3.6
No guideline available	15	4.8	4	1.9					11	39.3
Non-compliant with guideline	134	42.7	109	52.9	11	26.2	9	23.7	5	17.9
Not assessable	14	4.5	9	4.4	1	2.4	—	—	4	14.3
Appropriateness of prescription										
Appropriate	169	53.8	84	40.8	30	71.4	30	78.9	25	89.3
Inappropriate	132	42.0	112	54.4	10	23.8	8	21.1	2	7.1
Not assessable	13	4.1	10	4.9	2	4.8	—	—	1	3.6

CRRH, Central Regional Referral Hospital; ERRH, Eastern Regional Referral Hospital; JDWNRH, JDW National Referral Hospital; PGH, Phuentsholing General Hospital.

a Directed therapy where prescription was changed from empirical to directed therapy with microbiology culture or susceptibility report was available.

Among the prescriptions audited, the indication for use was documented in the prescriptions of 278 patients (88.5%) and a review or stop date was recorded in 102 (32.5%). The common indications for which antibiotics were prescribed included surgical prophylaxis (42; 13.4%), community-acquired pneumonia (39; 12.4%) and sepsis (26; 8.3%). Among patients receiving antibiotics for surgical prophylaxis, 22 (52.4%) had received antibiotics longer than 24 h.

**Figure 1.**
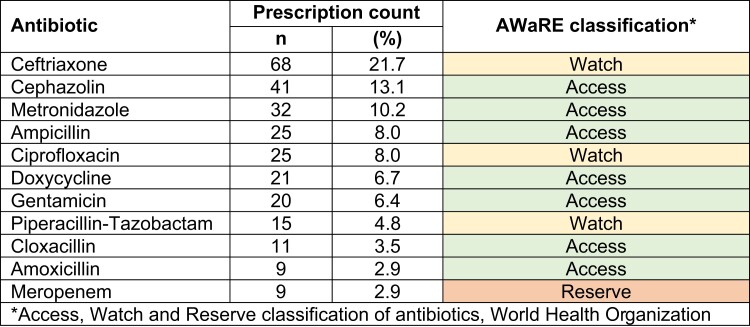
Top 10 antibiotics prescribed in patients admitted in the four major hospitals in Bhutan during the National Antimicrobial Prescribing Survey, June 2022 (*n* = 314 patients), including AWaRe classification. Amoxicillin and meropenem were joint tenth most frequently prescribed antibiotic.

**Figure 2.**
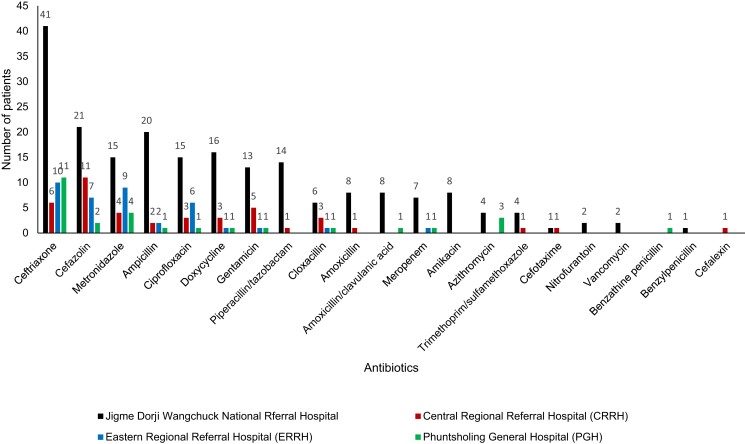
Frequencies of antibiotics prescribed in patients admitted in four major hospitals in Bhutan during the National Antimicrobial Prescribing Survey, June 2022 (*n* = 314 patients).

Across all hospitals, ceftriaxone (68; 21.7%), cefazolin (41; 13.1%) and metronidazole (32; 10.2%) were the most common antibiotics prescribed. There were 190 (60.5%) patients on one antibiotic, 112 (35.7%) on two antibiotics and 12 (3.8%) on three antibiotics. The top 10 commonly prescribed antibiotics and the WHO Access, Watch and Reserve (AWaRe) groups^12^ are shown in Figure 1. The details of the common antibiotics prescribed in the four hospitals are shown in Figure 2.

**Figure 3.**
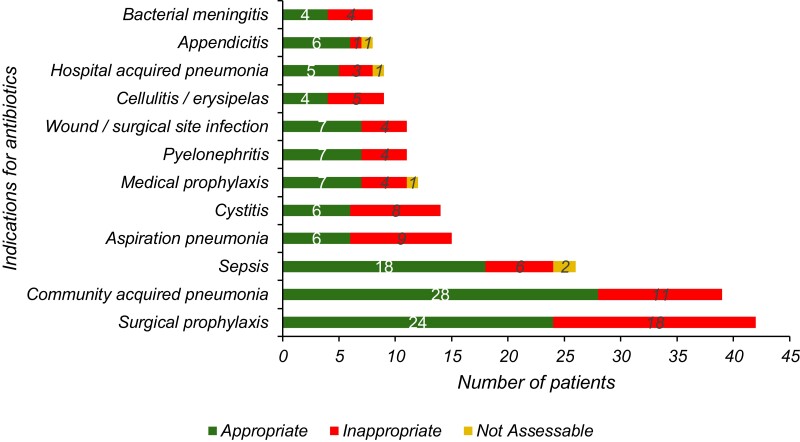
Indications for antibiotics in patients admitted in four major hospitals in Bhutan during the National Antimicrobial Prescribing Survey, June 2022 (*n* = 314 patients).

There were 134 prescriptions (42.7%) that were non-compliant with the National Antibiotic Guideline^10^ and there was no appropriate guideline available for 15 (4.8%) cases. There were only 26 patients (8.3%) in whom the prescription had changed from empirical to directed therapy based on microbiology findings. Overall, there were 169 patients (53.8%) who were receiving antibiotics that were inappropriate. The reasons for inappropriate prescriptions were the spectrum of antibiotic prescribed being too broad for the indication (56; 33.1%), incorrect duration (22; 13.0%), incorrect dose or frequency (20; 11.8%), antibiotic not indicated (20; 11.8%), spectrum too narrow (13; 7.7%) and incorrect route (1; 0.6%). The indications for antibiotics and the appropriateness of prescriptions are shown in Figure 3. The assessment of quality indicators for antibiotic prescriptions is shown in Table 1.

## Discussion

To our knowledge, this is the first survey in Bhutan that describes the prescription and use of antibiotics among patients admitted to four major hospitals that cater to large patient loads. The overall antibiotic prescription rate was 41.5%, with the highest proportion reported at the Central Regional Referral Hospital. The prevalence of antibiotic prescription is within the range of 37.3%–49.0% among inpatients reported in samples from within the region including India, Nepal and Malaysia.^13–15^ While studies from other settings report the highest prevalence of antibiotic prescription in ICUs, our data report the highest volume of patients on antibiotics in Internal Medicine wards.

In all hospitals, ceftriaxone was the most common antibiotic prescribed, followed by cefazolin, metronidazole and ampicillin. Ceftriaxone was one of the top antibiotics prescribed in studies reported from Nepal (32.7%), India (21.5%) and Malaysia (9.5%).^13–15^ It is important to note that Bhutan does not have a medical college and all its doctors/prescribers are trained in the countries in the region. Ceftriaxone is available only at secondary- and tertiary-level centres in Bhutan as per the National Essential Medicines List 2021.^16^ The overuse of ceftriaxone is a growing concern, with reports of increasing resistance to β-lactams and cephalosporins. Resistance to ampicillin (2.1%) and ceftriaxone (1.5%) was most the common in bacteriologically confirmed blood stream infections with *Escherichia coli*, according to the WHO Global Antimicrobial Resistance and Use Surveillance System (GLASS) report 2020 from Bhutan.^17^

The top indication for antibiotics was surgical prophylaxis, and the majority of these patients received antibiotics for durations longer than 24 h. This is a key area for attention as rates seem much higher than in other countries. The appropriateness of antibiotic choice and compliance with the National Antibiotic Guideline^10^ varied across hospitals. The majority of the prescriptions at the Central Regional Referral Hospital and Eastern Regional Referral Hospital were concordant with the national guidelines while JDW National Referral Hospital had much higher non-concordance observed. The JDW National Referral Hospital has a higher number of prescribers and trainee doctors, and receives many cases in critical conditions. The two regional referral hospitals have fewer number of prescribers and may also have a less complex patient case mix; however, further exploration on compliance to antibiotic guidelines is warranted.

There were several instances of poor documentation noted. In Bhutan, all medical records are on paper-based files that have open-ended space for prescribers to document clinical details. While good prescribing practices are recommended as per the standards of the Medical and Health Professionals Council and the Bhutan Medicines Rules and Regulations 2019,^18^ there are no established means to assess, audit and enforce these regulations on day-to-day practices. The Bhutan Food and Drug Administration has an established reporting mechanism for adverse drug reactions; however, history taking and documentation of drug allergy was inadequate for evaluation using the NAPS. With the Digital *Drukyul* project that will implement an electronic patient information system (ePIS), some of these concerns are expected to be addressed. It is recommended that there is a need for regular training of prescribers to adopt good prescribing practices.

This survey also identified the lack of collection of appropriate microbiology samples prior to the commencement of antibiotics. These four hospitals are equipped with microscopy and culture services that received upgraded facilities through the Fleming Fund project. The findings from this survey suggest a high proportion of missed opportunities for microbiology sampling that would have potentially guided more appropriate antibiotic therapy. Overall, microbiology-guided therapy was very low across all hospitals. This finding might have been due to this survey being a single point prevalence snapshot so had failed to follow through with how prescriptions are revised based on microbiology reports. However, it is recommended to provide continuing medical education to prescribers on the adoption and utilization of microbiology testing with a target of reducing inappropriate antibiotic prescriptions.

The adoption of the Australian NAPS tool was effective and user-friendly and there are plans to roll it out to other district hospitals. One of the advantages of this tool is the data analysis dashboard that helped hospitals establish baseline data and plan their target interventions. The findings from this study have identified the areas of interventions that can help promote stewardship initiatives to help meet the targets of the National Action Plan on Antimicrobial Resistance in Bhutan.^9^ Auditing and feedback about antimicrobial prescribing quality, with the use of this standardized and validated point prevalence survey tool, is now intended to be conducted at regular intervals to monitor improvement.

Activities for the prevention of AMR are now adopted at all levels of hospitals. The four hospitals have developed an annual workplan with specific activities under six areas of interventions: building leadership commitment; establishing accountability and responsibility of delivering the antimicrobial stewardship programme; key activities such as implementation of guidelines, conducting audits and establishing surveillance systems for hospital-acquired infections; education and training activities; monitoring and evaluation of activities; and reporting and feedback within the health facilities. The overall activities are a part of the hospital’s key performance activities and are reported to the national antimicrobial stewardship programme. The summary dashboard data generated from the NAPS have been given to the respective hospitals as their baseline data for targeted intervention within their facilities.

### Conclusions

The overall antibiotic prescription prevalence among inpatients in the four major hospitals in Bhutan is comparable to that from hospitals in the region. Opportunities to improve documentation, encourage use of the national antibiotic prescribing guidelines, and address surgical prophylaxis duration were highlighted. The Australian NAPS tool was successfully adopted in Bhutan and is planned to be used in other hospitals to support antimicrobial stewardship.
